# WHO and Global Health Monitoring: The Way Forward

**DOI:** 10.1371/journal.pmed.1000373

**Published:** 2010-11-30

**Authors:** J. Ties Boerma, Colin Mathers, Carla Abou-Zahr

**Affiliations:** 1 World Health Organization, Geneva, Switzerland; 2 Geneva, Switzerland

## Abstract

Ties Boerma and colleagues from WHO describe the agency's work and future in health indicator monitoring, as part of a cluster of *PLoS Medicine* articles on global health estimates.

**Summary Points**

- The growing demand for reliable data to monitor progress in health has highlighted the need to strengthen the way estimates for health indicators are generated.
- Global health estimation work should meet agreed standards of transparency, scientific rigour, and accessibility. Current work under the aegis of the World Health Organization (WHO) and other United Nations agencies needs to be strengthened to meet these standards, in close collaboration with academic institutions.
- WHO is well positioned to continue to play a lead role in statistical estimation work because of its constitutional mandate, its accountability to member states, its ability to mobilize global expertise, and its unique position to generate productive interactions between global monitoring and country information systems.
- Countries would benefit most from global collaborative efforts that include support for data collection, sharing of data, development of scientific methods of estimation, publication of estimates, development of estimation tools, and country capacity strengthening.

*This article is part of a cluster of five articles on global health estimates.*

## Four Essential Criteria for Health Indicator Estimates

Global, regional, and country statistics on population and health indicators are important for assessing development and health progress and for guiding resource allocation, but data are often lacking, especially in low- and middle-income countries. To fill the gaps, statistical modeling is frequently used to produce comparable health statistics across countries that can be combined to produce regional and global statistics. Modeling brings together data from different sources and uses a range of statistical techniques to correct for biases, impute values where data are lacking, and predict current values for key health indicators. Estimation work, whether it be conducted under the aegis of an agency like the World Health Organization (WHO) or in an academic institution, should meet agreed standards of transparency, scientific rigour, and accessibility. Building upon WHO-issued internal guidelines for producing global, regional, and country estimates, four essential criteria can be identified [1],[2]. For each we offer the WHO perspective on current status and scope for improvement in work on estimates for health indicators.

### 1. Public Access to Core Input Data

All estimates should be based on publicly accessible and comprehensive databases. WHO and other UN agencies maintain multiple databases containing country statistics on an array of indicators, collected through household surveys, censuses, civil registration systems, clinical reporting systems, disease surveillance, administrative sources, and research. These data are usually aggregated at the national level. Sharing of individual data (micro data) is largely limited to data from censuses and surveys collated by international survey programmes.

There is scope to enhance the completeness, timeliness, and quality of and public access to the international databases. Currently they suffer from underinvestment, at both country and global levels. Eight international health agencies recently called for enhancement of access to country- and global-level data, statistics, and metadata, with appropriate security and confidentiality measures [3]. Improving the quality and accessibility of databases should be a joint effort of countries, academic institutions, and UN agencies. WHO is well positioned to facilitate access to data and metadata, provided adequate resources are invested in building country capacities for data management, archiving, updating, and maintenance.

### 2. Transparency, and Use of and Access to, Sound Statistical Methods

All estimates should be developed using transparent and scientifically sound methods, reviewed by independent technical experts, and made publicly available, preferably through freely available publication in peer-reviewed scientific journals. Adjustments to incomplete or inaccurate data need to be explained and justified. There should be clear documentation of decisions regarding choice of statistical regression analyses used to impute missing values and predict estimates from multiple data points. Choices regarding covariates used to predict missing values should be described and justified. There should also be a systematic comparison of the performance of different models and a specification of the precision of the estimates, often referred to as uncertainty range. Estimates should be replicable at the time of publication; that is, raw data, data adjustments, programming code, covariate data, and demographic envelopes should be easily accessible.

Currently, neither the UN agencies nor academic institutions have fully achieved this standard. While the use of advanced statistical methods is desirable, in-country replicability is equally important. Estimation methods that are developed into user-friendly country tools are more likely to be used for decision making. As stated by the heads of eight leading health agencies [3]:

[C]omparable estimates for key health indicators, such as child and maternal mortality or immunization coverage, should be made on the basis of the best possible data with the best possible methods in a comprehensible, transparent manner which allows reproduction of the estimates at country and global levels. Global technical debates are useful to improve methods and estimates but should be conducted in a manner that minimizes confusion among health planners and programmers.

### 3. Review by an Independent Expert Group

The methods and results of the estimation process should be reviewed by an advisory group of independent experts. Currently, methodological advances published by academic institutions outside of the expert groups are reviewed and taken into account to improve WHO/UN estimation work. In addition, the reviews should also consider other elements such as adherence to WHO classification standards, consistency with other health statistics (e.g., all-cause mortality levels), internal consistency of statistics (e.g., incidence and prevalence estimates for the same disease), use of WHO and UN standard estimates where relevant (e.g., population estimates and health expenditure estimates), and comparability of estimates across populations or time.

There are several successful models of this within the UN system. For example, the WHO–United Nations Children's Fund (UNICEF) Child Health Epidemiology Reference Group (CHERG) is led by Johns Hopkins University, holds a grant from the Bill & Melinda Gates Foundation, and develops estimates of causes of death in children under five. The Technical Advisory Group of the Inter-Agency Group for Child Mortality Estimates (IGME) advises WHO, UNICEF, World Bank, and the UN Population Division on annual updates of estimates of child mortality rates and is supported by UNICEF and WHO core funds. The Joint United Nations Programme on HIV/AIDS (UNAIDS) Reference Group on Estimates, Modeling, and Projections, funded by UNAIDS, has published extensively on methods and tools for estimating incidence, prevalence, and mortality due to HIV/AIDS, but has also invested in tool development and capacity building in order to build country ownership of the results.

While experts generally provide their time free of charge, sustained funding for convening and supporting the expert groups is critical. Funding requirements depend on the scope and frequency of the estimation work, but it is clear that the UN requires commensurate levels of investment to meet the demand for more frequent updates and greater transparency.

Expert group members may actively engage in the actual estimation work, either funded independently or through the UN, as long as all criteria for transparency are adhered to.

### 4. Country Engagement

In line with a WHO Executive Board resolution (EB107.R8 in 2001), public release of country estimates should be preceded by consultation with WHO Member States. The consultation provides countries with an opportunity to comment on methods and data sources and to contribute updated input data and typically lasts two months. In the process of consulting with countries prior to the release of estimates, WHO does not give in to political pressures to report particular values but may publish its own comparable estimates and the country-reported numbers side by side, as was done for maternal mortality in 2010 [4]. Such discrepancies are increasingly leading to efforts by countries, often in collaboration with WHO, to improve the underlying availability and quality of data for the estimates.

To be most successful, the consultation process should be accompanied by user-friendly tools and country analytical capacity strengthening. The significant resources required to build adequate country estimation capacity have been mobilized in only a few health areas, the most notable of which is HIV/AIDS. User-friendly tools and multiple rounds of training were needed to create sufficient country capacity to take ownership of the estimates.

## Options for WHO's Work in Estimation

The challenge for WHO, UNICEF, and other UN agencies is to decide whether to continue developing estimates for key health indicators, and to determine what kinds of relationships to pursue with academic institutions that have begun to develop their own estimates. The advent of new actors in the area of global estimation has stimulated WHO to reexamine its own activities in this area and to consider how they might be modified. Three options present themselves for the future work of WHO.

### Option 1: Strengthening the Current WHO/UN Model

In scenario 1, the relevant UN agencies continue to work together in an inter-agency group, supported by expert groups composed of leading technical experts. The UN agencies provide the financial resources for meetings and consultancy work and are responsible for the databases. Since the UN does not pay expert group members (only travel expenses are compensated), the success of this model depends on the continuing willingness of academic experts to give time to the UN on a pro bono basis.

Critical factors are maximum transparency at all stages and increased investment in the estimation procedure. This would result in high-quality, up-to-date databases (with metadata); availability of expert groups with broad-based participation of leading academics and a budget to commission methodological work; development of user-friendly estimation tools and training packages; and a major multi-round effort to strengthen country capacity to produce estimates. All relevant academic institutions would contribute to data sharing, to methods and tools development, and to working together to develop the best estimates.

### Option 2: Creating an Independent Agency Linked to UN Agencies

In scenario 2, an independent body linked to WHO evaluates the estimates process at WHO. This arrangement could resemble existing arrangements where some UN agencies already have established entities that carry out independent evaluations of agency activities. The World Bank's Independent Evaluation Group, an independent unit within the World Bank that evaluates World Bank projects, reports directly to the upper echelons of the organization. The United Nations Educational, Scientific, and Cultural Organization (UNESCO) Institute for Statistics is the statistical branch of UNESCO, hosted by the University of Montreal, Canada, and reporting to a board consisting of statistical experts representing different regions and international organizations.

### Option 3: Academic Institutions Taking the Lead in the Development of Global Estimates

In scenario 3, academic institutions take the lead in producing regular updates of estimates. The UN system's role would be limited to data collection and dissemination, and to technical advice. Moving global health monitoring away from the UN system to an independent health monitoring entity was recommended by Murray et al. [5]. However, at present, only the Institute for Health Metrics and Evaluation at the University of Washington, with generous funding from the Bill & Melinda Gates Foundation, has demonstrated the interest and capability to produce multiple estimates independent of the UN system.

## Our Preferred Option

The growing demand for reliable data to monitor progress in health has highlighted the need to reinvigorate and strengthen the way estimates are generated and to address the underlying data gaps in countries, without running into protracted academic debates. The production and dissemination of health statistics for health action at the country, regional, and global levels are core WHO activities mandated by the Member States in the organization's constitution. We see no reason why WHO should renounce this core activity in response to the parallel estimation activities of academic institutions. There are several reasons for this position:

- WHO statistics carry great weight in national and international resource allocation, policy-making, and programming because of WHO's reputation as an entity that is unbiased (impartial and fair), global (having a worldwide remit and responsibility), and technically competent (drawing on leading research and policy institutions and individuals).
- International agencies such as WHO have a long history of global-, regional-, and country-level action and are constituted by representatives of national governments. This implies ongoing and time-unlimited commitment to health and development. By contrast, the activities of academic institutions in relation to estimation are mainly funded by external donors and liable to be dependent on the availability of resources. Academics are likely to lose interest in global estimation should funding levels decline or scientific publication become more difficult.
- WHO is accountable to the Member States and committed to working with them to enhance capacities both to measure health and mortality and to implement interventions to address health-related problems. Academic institutions have no such accountability and are rarely interested in or capable of providing such country support on a continuous basis.
- The UN system is better positioned than academic institutions to generate productive interactions between global monitoring efforts and country information systems. Estimates of the HIV/AIDS epidemic are generated by countries themselves using the standardized methods developed through the UNAIDS Epidemiology Reference Group. In addition to empowering countries and stimulating the use of data for health action, this approach has demonstrated the importance of solid surveillance systems. Thus, country capacity building is producing not only better country data but also better global monitoring.

This is not a call for complacency or business as usual. On the contrary, the activities of academics in estimation of health-related indicators have been of tremendous value in terms of the development of innovative statistical techniques and the critical appraisal of available data. WHO and the UN system can greatly benefit from such innovations and should make every effort to incorporate those of value into their own work. We feel that countries would benefit most from a collaborative effort of all lead experts that includes collection and sharing of data, development of scientific methods of estimation, publication of estimates, development of user-friendly estimation tools, and country capacity strengthening.

## Abbreviations

UNAIDS: the Joint United Nations Programme on HIV/AIDS
UNESCO: United Nations Educational, Scientific, and Cultural Organization
UNICEF: United Nations Children's Fund
WHO: World Health Organization
